# Anhedonia and suicidal ideation in young people with early psychosis: findings from a 2-year Italian follow-up study.

**DOI:** 10.1192/j.eurpsy.2024.1638

**Published:** 2024-08-27

**Authors:** L. Pelizza, M. Poletti, A. Raballo, A. Di Lisi

**Affiliations:** ^1^Università di Bologna, Bologna; ^2^AUSL-IRCCS di Reggio Emilia, Reggio Emilia, Italy; ^3^Università della Svizzera Italiana, Lugano, Switzerland; ^4^Alma Mater Studiorum University of Bologna, Bologna, Italy

## Abstract

**Introduction:**

Hedonic deficits have been extensively studied in schizophrenia, but little is known about their association with suicidal ideation in early psychosis. Along the clinical staging of psychosis, also Ultra-High Risk (UHR) individuals are characterized by hedonic deficits, which are currently considered as putative predictors of both psychosis conversion and poor social/role functioning.

**Objectives:**

The aim of this research was to examine the relationship between anhedonia and suicidal thoughts across a 2-year follow-up period in people with First Episode Psychosis (FEP) and at Ultra High Risk (UHR) of psychosis.

**Methods:**

Ninty-six UHR and 146 FEP, aged 13–35 years, completed the Comprehensive Assessment of At-Risk Mental States (CAARMS) and the Beck Depression Inventory-II (BDI-II). The BDI-II “Anhedonia” subscale score to assess anhedonia and the CAARMS “Depression” item 7.2 subscore to measure depression were used across the 2 years of follow-up. Hierarchical regression analyses were performed.

**Results:**

No difference in anhedonia scores between FEP and UHR individuals was found. In the FEP group, a significant enduring association between anhedonia and suicidal ideation was found at baseline and across the follow-up, independent of clinical depression. In the UHR subgroup, the enduring relationship between anhedonia and suicidal thoughts were not completely independent from depression severity.

**Conclusions:**

Anhedonia is relevant in predicting suicidal ideation in early psychosis. Specific pharmacological and/or psychosocial interventions on anhedonia within specialized EIP program could reduce suicide risk overtime.

**Disclosure of Interest:**

None Declared

